# NeuroD1 Regulated Endothelial Gene Expression to Modulate Transduction of AAV-PHP.eB and Recovery Progress after Ischemic Stroke

**DOI:** 10.14336/AD.2023.1213

**Published:** 2023-12-20

**Authors:** Xiaosong He, Xin Wang, Hui Wang, Tao Wang, Fuhan Yang, Yuchen Chen, Zifei Pei, Yuting Bai, Wen Li, Zheng Wu, Gong Chen

**Affiliations:** ^1^Emergency Department, Department of Neurology of the Second Affiliated Hospital of Guangzhou Medical University, Guangzhou, China.; ^2^Department of Neurology of the Sixth Affiliated Hospital of Guangzhou Medical University, Department of Human Anatomy in School of Basic Science of Guangzhou Medical University, Key Laboratory of Neurogenetics and Channelopathies of Guangdong Province and the Ministry of Education of China, Guangzhou, China.; ^3^Department of Biology, Huck Institutes of Life Sciences, Pennsylvania State University, University Park, PA 16802, USA.; ^4^Department of Neurology, Affiliated ZhongDa Hospital, School of Medicine, Southeast University, Nanjing, China.; ^5^GHM Institute of CNS Regeneration, Jinan University, Guangzhou, 510632, China.

**Keywords:** AAV, intravenous gene therapy, ischemic stroke, NeuroD1, vascular injury, neural repair

## Abstract

AAV-PHP.eB depends on endothelial cells to highly transduce the central nervous system (CNS) and is widely used for intravenous gene therapy. However, the transduction profile and therapeutic efficiency after endothelial cell injury such as ischemic stroke is largely unknown. In this study, we tested the transduction profiles of AAV-PHP.eB and developed intravenous NeuroD1 gene therapy to treat ischemic stroke in mice. We found that AAV-PHP.eB-GFP control virus crossed the BBB and infected brain cells efficiently in normal brain. However, after stroke, AAV-PHP.eB-GFP control virus was highly restricted in the blood vessels. Surprisingly, after switching to therapeutic vector AAV-PHP.eB-NeuroD1-GFP, the viral vector successfully crossed blood vessels and infected brain cells. Using Tie2-cre transgenic mice, we demonstrated that NeuroD1 regulated endothelial gene expression to modulate AAV-PHP.eB transduction. Following the changes of signaling pathways in endothelial cells, NeuroD1 effectively protected BBB integrity, attenuated neuroinflammation, inhibited neuron apoptosis and rescued motor deficits after ischemic stroke. Moreover, NeuroD1 over-expression in brain cells further promoted neural regeneration. These results indicate that intravenous gene therapy using AAV-PHP.eB for ischemic stroke differs from intracranial gene therapy and NeuroD1 intravenous delivery using AAV-PHP.eB efficiently rescue both vascular damage and neuronal loss, providing an advancing therapeutic treatment for stroke.

## INTRODUCTION

Stroke remains a leading cause of death and disability worldwide despite decades of efforts to find suitable treatment. Significant progress has been made to treat acute ischemic stroke, but the overall therapeutic efficacy remains relatively low [1]. The success of neuroprotection in animal stroke models had previously raised high hope, but the subsequent failures of clinical trials in stroke patients have led to some pessimism in the stroke field. One may attribute the failure to the complex pathophysiology after stroke and gaps between stroke in humans and animal models [2]. However, since stroke leads to intertwined damages in the brain, including dysfunctional blood vessels, severe neuroinflammation, and irreversible neural injury, simply rescuing one particular part might not be enough to have significant effect. A multitarget therapy after stroke might provide more comprehensive improvements.

Emerging new technologies, such as gene therapy, may provide new tools to aim at multiple targets simultaneously for stroke treatment. Adeno-associated virus (AAV) has been proven to be an ideal viral vector to deliver transgenes due to its relatively low toxicity and low immune reactions in humans. We have recently developed NeuroD1 AAV-based neuroregenerative gene therapy to treat various neurological disorders, including stroke, Huntington's disease, traumatic brain injury, and spinal cord injury [3-7]. We and others have demonstrated that NeuroD1 can convert lineage-traced astrocytes into neurons [8, 9], particularly after using enhancer to increase NeuroD1 expression levels [10, 11]. The success of such AAV-based gene therapy relies heavily on the efficiency of AAV entering the brain parenchyma and overexpressing transgenes in the targeted brain cells. Recently, a novel AAV serotype, AAV-PHP.eB has been developed to effectively cross the blood-brain barrier (BBB) and transduce the central nervous system (CNS) with high efficiency [12-14]. This breakthrough provides a non-invasive tool for gene manipulation within the CNS. The process of AAV-PHP.eB transduction encompasses distinct stages: initial attachment to endothelial cells, subsequent endothelial cell transcytosis, and eventual transgene expression in brain cells. Notably, the efficiency of AAV-PHP.eB transduction in brain cells is closely linked to the properties of endothelial cells [15-17]. It is widely recognized that numerous central nervous system (CNS) disorders, such as stroke, traumatic brain injury, seizures, and Alzheimer's disease (AD), are characterized by substantial alterations in the gene expression of endothelial cells and a compromised blood-brain barrier (BBB) function [18, 19]. However, whether the endothelial gene expression changes under pathological conditions could impact the transduction profile and therapeutic efficacy of gene therapy using AAV-PHP.eB are not well studied.

In this study, we first present data showing that ischemic stroke induced endothelial cell gene expression changes leads to a specific spatial-temporal endothelial transduction profile of control AAV-PHP.eB-EF1α-GFP delivered through intravenous injection in a mouse middle cerebral artery occlusion (MCAO) model. Then, we found that, by regulating endothelial cell gene transcription, intravenous delivery of AAV-PHP.eB-EF1α-NeuroD1-GFP resulted in the exit of the AAV vector from blood vessels and wide infection of the brain cells and displayed an improved ability in rescuing BBB leakage and neuron loss after MCAO. The utilization of AAV-PHP.eB intravenous gene therapy may provide an effective approach in rescuing neurovascular impairment post ischemic stroke.

## MATERIALS AND METHODS

### Animals

Wild-type C57BL/6 (JAX Laboratories) mice were used in this study. Tie2-cre mice were ordered from Gempharmatech Co. Ltd. Animals were housed under standard conditions. All animal handling was performed to minimize suffering. Animal procedures were performed following the Animal Protection Guidelines of the US National Institutes of Health [20], and all experimental protocols were approved by the Pennsylvania State University’s Institutional Animal Care and Use Committee (IACUC) and Guangzhou Medical University Institutional Animal Care and Use Committee.

### Mouse model of MCAO

In the study, 8-10-week-old male mice weighing 23-27g were used. Mouse model of MCAO was performed as previously reported [21]. Briefly, adult mice were anesthetized by intraperitoneal injection of ketamine/xylazine (100 mg/kg ketamine; 12 mg/kg xylazine). Then, the left external carotid artery (ECA), internal carotid artery and common carotid artery (CCA) were exposed, a 6-0 suture (Doccol, Massachusetts, USA) was inserted from the external carotid artery (ECA) into the internal carotid artery (ICA) to occlude the origin of middle cerebral artery (MCA). Reperfusion was performed by withdrawn the suture 40 min after the occlusion. The cerebral blood flow (CBF) was measured before surgery, after the occlusion and reperfusion used a Laser doppler instrument (Moor instrument, Devon, UK). The CBF was sharply down to 10% after occlusion and return to 70% of baseline was recognized as success of MCAO. After surgery, the mice were randomly assigned to the following treatment groups: AAV-PHP.eB-EF1α-GFP group and AAV-PHP.eB-EF1α-NeuroD1-GFP.

### AAV Virus Production

Recombinant AAV9 and AAV-PHP.EB was produced in 293AAV cells (Cell Biolabs, San Diego, USA). Polyethylenimine (PEI, linear, MW 25,000) was used for transfection of triple plasmids: pAAV, pAAV-RC and pHelper (Cell Biolab). Three days post transfection, cells with their medium were collected and centrifuged. The cells were lysed by placing it alternately in -80? cooled ethanol and 37? water bath four times. Lysate was purified by centrifugation at 54,000 rpm for 1 h in discontinuous iodixanol gradients. The virus-containing layer was extracted, and viruses were concentrated by Millipore Amicon Ultra Centrifugal Filters. Virus titers determined by QuickTiter AAV Quantitation Kit (Cell Biolabs). The titers were 1.2 × 10^12^ GC/mL for hGFAP::Cre, 1.6 × 10^12^ GC/mL for CAG::FLEX-P2A-mCherry, 1.3 × 10^13^ GC/mL for EF1-a-P2A-GFP and 1.2×10^13^ GC/mL for EF-1a-NeuroD1-P2A-GFP. The viruses AAV-PHP.eB-CAG::Flex-P2A-GFP and AAV-PHP.eB- CAG::Flex-NeuroD1-P2A-GFP used for Tie2-Cre mice were ordered from Guangzhou Packgene Co. Ltd ( Guangzhou, China) and the titers were both 1.0 × 10^13^ GC/mL.

### Virus Injection

For intracranial virus injection, mice were anesthetized by intraperitoneal injection of ketamine/xylazine (100 mg/kg ketamine, 12 mg/kg xylazine), and placed in a stereotaxic apparatus. A 0.5-mm bone hole was drilled by a hand drill (Fine Science Tool, Foster City, CA). Viruses were slowly injected into the left striatum (AP = - 0.02 mm, ML = - 2.5 mm, DV = 3 mm relative to the bregma) at the speed of 0.1 μl/min. After injection, the needle was kept for additional 5 min before fully withdrawn. The bone hole was then sealed by bone wax and the skin was sutured.

For intravenous virus injection, the virus (1.3×10^13^ gc/ml, total 20μL) was diluted into 100ul D-PBS. After reperfusion in MCAO mice or fully anesthetized in normal mice, the virus was retro-orbitally injected into the vein.

### RNA Extraction

RNA extractions were performed using the Macherey-Nagel NucleoSpin® RNA kit (Macherey-Nagel, Duren, Germany) according to the manufacturer. The concentration and purity of the samples were measured using NanoDropTM 2000 spectrophotometers (Thermo Fisher Scientific, Waltham, USA).

### RT-PCR and RNA-Sequencing Analysis

For cDNA synthesis, RNAs were mixed with Quanta Biosciences qScript cDNA supermix and incubated at 25°C for 5 min, 42°C for 30 min, 85°C for 5 min. The primers for real-time qPCR were designed using Applied Biosystems Primer Express software and synthesized in IDT. Primers used in the current study were as listed, GAPDH Forward: GGAGCGAGACCCCACTAACA, Reverse: ACATACTCAGCACCGGCCTC; IL6 Forward: TTCCATCCAGTTGCCTTCTTG, Reverse: CATTTCCACGATTTCCCAGAG; MMP9 Forward: CTGGACAGCCAGACACTAAAG Reverse: CTCGCG GCAAGTCTTCAGAG; IFNγ Forward: ATGAACG CTACACACTGCATC, Reverse: CCATCCTTTTGCC AGTTCCTC. For qRT-PCR, each reaction, 6.25 μl SYBR Green Supermix (Quanta Biosciences PerfeCTa, ROX), 2 μl cDNA, and 3.75 μl water were mixed well and loaded in a 96-well plate (Applied Biosystem Inc, Waltham, USA).

For RNA-seq, RNA quality check, mRNA enrichment, library construction, and single-end 50bp sequencing with HiSeq 3000 were performed at the UCLA Technology Center for Genomics and Bioinformatics. To get the expression profile in normal cortex, we download our previous sequencing data from NCBI (GSE135981). Then, Quality checking of the raw data was done using FastQC (v.0.11.3). The filtered reads were aligned against human reference genome hg38 using HISAT2 (v.2.0.1) and summarized using feature counts (v.1.5.0). Differential expression analysis was processed using DESeq2 (v.1.16.1) and genes with more than 2-fold differences, basemean > 80 and adjusted *p* < 0.05, were called deferentially expressed genes (DEGs) [22].

For Drug-seq, Tie2-cre mice underwent AAV-PHP.eB-CAG-Flex-GFP or AAV-PHP.eB-CAG-Flex-NeuroD1-P2A-GFP retrobital injection, the tissue including cortex and striatum was collected 14 days post virus injection. The brain endothelials were collected by magnetic cell sorting (MACS, Miltenyi Biotec). 50000 endothelial cells collect from three mice were labeled by Barcode, PCR, library construction, and sequencing were performed at Epibiotek Co. LD (Guangzhou, China) as previously reported [23]. Reads were aligned to the mouse Ensemble genome GRCm38 using bowtie2 aligner (v 2.3.5.1) under parameters: “-rna-strandness RF”. The reads mapped the genome were calculated using featureCounts (v1.6.3). Differential gene expression analysis was performed using the DESeq R-package. Genes with more than 2-fold differences, baseMean > 0.5 and adjusted *p* < 0.05, were called deferentially expressed genes (DEGs).

### Bederson Score and Open field test

The Bederson Score was performed by a researcher who was blinded by the experiment design. The Bederson score was modified for use in mice (grade 0 no observable neurological deficit; grade 1 unable to extend the contralateral forelimb; grade 2 flexion of the contralateral forelimb; grade 3 mild circling to the contralateral side; grade 4 preserve circling; and grade 5 falling to the contralateral side) [24].

For the open field test, the mice were put into the center of an arena with 50*50*50cm, and allowed to freely move in 5 min, and then the trial in another 5 min was recorded by a digital camera using the EthoVision XT video tracking system and software (Noldus, Littletown, USA ). The total moving distance and average speed in a trial were calculated.

### MRI scanning and Infarct volume measurement

Imaging was carried out on a Varian 7 Tesla, 31 cm horizontal bore system equipped with a 12-cm inner diameter actively shielded gradient system (400 mT/m). T2-weighted images to visualize infarction were acquired using MSME sequences. Parameters were echoing time/repetition time 2500 ms/29 ms, 4 averages, matrix size of 192 × 192 giving an in-plane resolution of 78 × 78 μm, slice thickness of 4 mm, field of view 15×15 mm. Lesion size was measured on T2-weighted images using ImageJ (v1.45 r, NIH).

### Immunohistochemistry

Mice were fully anesthetized with avertin (200 mg/kg body weight). After then, mice were perfused with saline and sequenced with 4% paraformaldehyde fixation solution through left ventricle. After post-fixation in 4% paraformaldehyde overnight, the brain was coronal sectioned to a 50μm thick slice with a viabrotome (Leica, Wetzlar, Germany). To test the ZO-1 and Occludin expression, the brains were quickly freezed with isopentane without a post fixation in 4% para-formaldehyde, and sliced in the cryostat instrument (Leica, Wetzlar, Germany) at the thickness of 20 μm. For immunofluorescence staining, sections were rinsed 3 times by PBS for 5 min, blocked by 5% normal donkey serum for 60 min, incubated with primary antibodies at 4°C more than 24 hours. The primary antibodies used are below: Chicken anti-GFP (1:1000, Ab1397, Abcam), rabbit anti-GFAP (1:500, AB5541, Millipore), chicken anti-GFAP (1:1000, ab5541, Millipore), rabbit anti-IBA1 (1:600, 019-19741, Wako), goat anti-IBA1 (1:500, ab5076, Abcam), rat anti-Ly6C (1:500, ab24973, Abcam), rat anti-Ly6A (1:200, 557403, BD), rabbit anti-NeuN (1:1000, ABN78, Millipore), mouse anti-NeuroD1 (1:1000, ab60764 Abcam), rat anti-RFP (1:500, ABIN964932, abonline), rat anti-CD31 (1:300, 552074 BD), mouse anti-ZO-1 (1:200, 33-9100, Invitrogen), mouse-anti-Occudin (1:200, 33-1500, Invitrogene). Another day, sections were incubated with secondary antibodies for 120 min at room temperature. To ensure the specificity of the primary antibody in our experiment, we conducted control sections where only the secondary antibody was incubated. For terminal deoxynucleotidyl transferase dUTP nick end labeling (TUNEL) and NeuN double staining, TUNEL staining was first performed according to the manufacture (12156792910, Roche), and NeuN staining was performed as described above. All immunostaining data was analyzed by a blinded investigator and images were taken by an Olympus confocal microscope (Olympus, Tokyo, Japan). Images were acquired at 200× (whole brain images) or 400× (high resolution images). Two sections from each brain and four fields in one section were randomly selected for photograph. Then, all images were systematically processed and analyzed using Olympus Microscope Solutions Imaging Software (FV31S-DT) or Image J (U.S. National Institutes of Health).

### Western blot analysis

7 days after MCAO, mice were anesthetized as described above. Brains were quickly removed to a cooled brain mold, and then cut into four coronal sections with 2 mm apart; the second section including ischemic core was collected and divided into cortex and striatum. For protein extraction, tissues were first homogenized by ultrasonic on ice, and then centrifuged. Equal amount of total protein (40μg) was loaded on 10% (W/V) SDS-PAGE and transferred to PVDF membrane (Whatman, Piscataway, USA). For immunobloting, the membrane was blocked with 5% non-fat milk in Tris buffered saline with tween-20 (TBST) for 120 min at room temperature and subsequently with anti-ZO-1 (1:1000) or anti-Occludin antibody (1:1000) and anti-GAPDH (1:10000, sab5600208, sigma) antibody at 4ºC overnight. The membrane was washed in TBST and incubated with appropriate anti-rabbit or anti-mouse IgG (LI-COR) in TBST for 120 min at room temperature. Immunoreactivity was detected using the LI-COR Odyssey imaging system according to the company’s instructions. Immunoblot band intensities were quantified using Image J software.

### Statistical analysis

Statistical analysis was carried out with Prism Graphad 8 (GraphPad, Jolla). All results were expressed as Mean ± SD. Sharpiro-Wilk test was used to determine the normality of the data. Survival analysis was conducted by Gehan-Breslow-Wilcoxon test. Comparisons between two groups were made by Upaired t test (normal distribution) or Mann-Whitney test (non-normal distribution). One-way or Two- way ANOVA with Bonferroni post hoc test multiple comparisons was used for the analysis of multi groups (normal distribution). *p* <0.05 was considered statistically significant. All n and *p* values together with the statistics test were indicated in the figure legend and/or results section.

**Figure 1.**
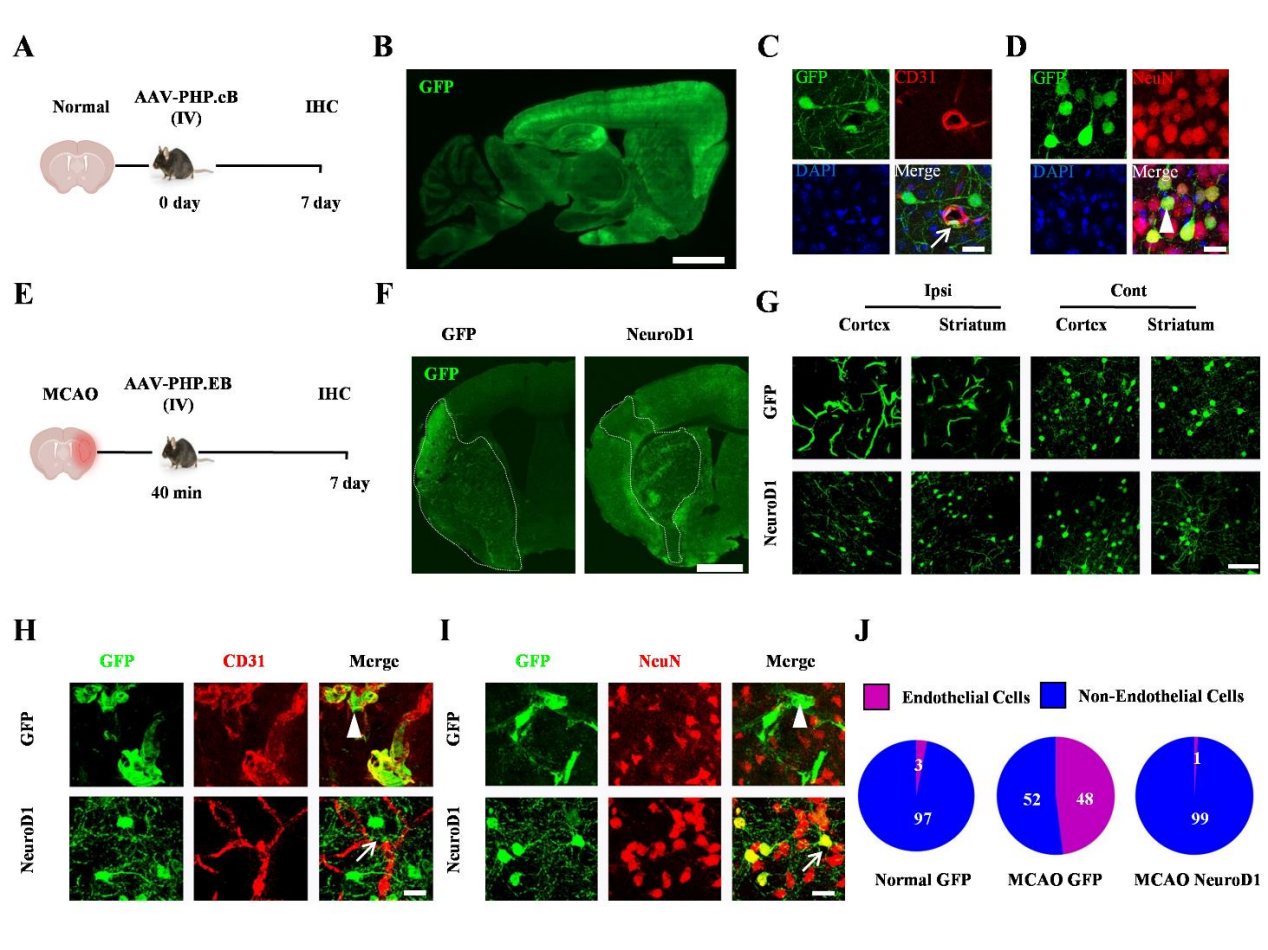
**AAV-PHP.eB displays specific transduction patterns after MCAO. (A)** Representative schematic of the experimental design for the control condition. AAV-PHP.eB was intravenously injected in normal adult C57BL/6 mice. Mice were sacrificed at 7 days post injection (dpi). Immunohistochemistry (IHC) was performed to investigate the viral transduction of mouse brains. **(B)** A sample image illustrating the global transduction by control AAV-PHP.eB-EF1α-GFP at 7 dpi, indicated by the IHC of GFP in sagittal brain sections from the control mice. Bar = 1 mm. **(C-D)** Representative high magnification images showing that a small number of AAV-PHP.eB-EF1α-GFP transduced cells (GFP, green, C) were endothelial cells (CD31, red, C) in control mice. But most transduced cells (GFP, Green, D) were neurons (NeuN, red, D) at 7 dpi. The arrow indicated the transduced endothelial cell. The arrowhead indicated the transduced neuron. Bar = 20 μm. **(E)** Representative schematic of the experimental design for MCAO condition. Reperfusion was performed by withdrawing the suture 40 min after the occlusion. AAV-PHP.eB was intravenously injected right after reperfusion and mice were sacrificed at 7 days post MCAO surgery and injection. IHC was performed to investigate the infarction and viral transduction. **(F)** Low magnification images showing the transduction of AAV-PHP.eB-EF1α-GFP (GFP, left) and AAV-PHP.eB-EF1α-NeuroD1-GFP (NeuroD1, right) in MCAO mice at 7 dpi. Dashed lines indicated the infarct zone. Note that AAV-PHP.eB-EF1α-GFP transduced endothelial cells mainly located in the infarct area and the transduced neurons were in the peri-infarct area. However, most AAV-PHP.eB-EF1α-NeuroD1-GFP transduced cells were neurons, which exhibited a wide distribution involving both infarct and peri-infarct areas. Bar = 1 mm. **(G)** High magnification images showing the AAV-PHP.eB-EF1α-GFP (GFP, upper panel) or AAV-PHP.eB-EF1α-NeuroD1-GFP transduced cells (NeuroD1, lower panel) in ipsilateral side (Ipsi, left) and contralateral side (Cont, right) from cortex or striatum of MCAO mouse brains. Note that most of the AAV-PHP.eB-EF1α-GFP transduced cells in the ipsilateral side of MCAO were endothelial cells based on the morphology. **(H-I)** Representative high magnification images showing the AAV-PHP.eB-EF1α-GFP transduced cells (GFP, green) were endothelial cells (CD31, red, H) not neurons (NeuN, red, I) in the ipsilateral side of MCAO (GFP, upper panel). In contrast, the AAV-PHP.eB-EF1α-NeuroD1-GFP transduced cells (GFP, green) were neurons (NeuN, red, I) rather than endothelial cells (CD31, red, H) in the infarct area of MCAO (NeuroD1, lower panel). Arrows indicated the transduced endothelial cells. Arrowheads indicated the transduced neurons. Samples were at 7 dpi. Bar = 20 μm. (J) Quantification of endothelial and non-endothelial cells among all transduced cells, n = 3 in normal mice, n = 5 in MCAO/GFP group, n = 6 in MCAO/NeuroD1 group.

**Figure 2.**
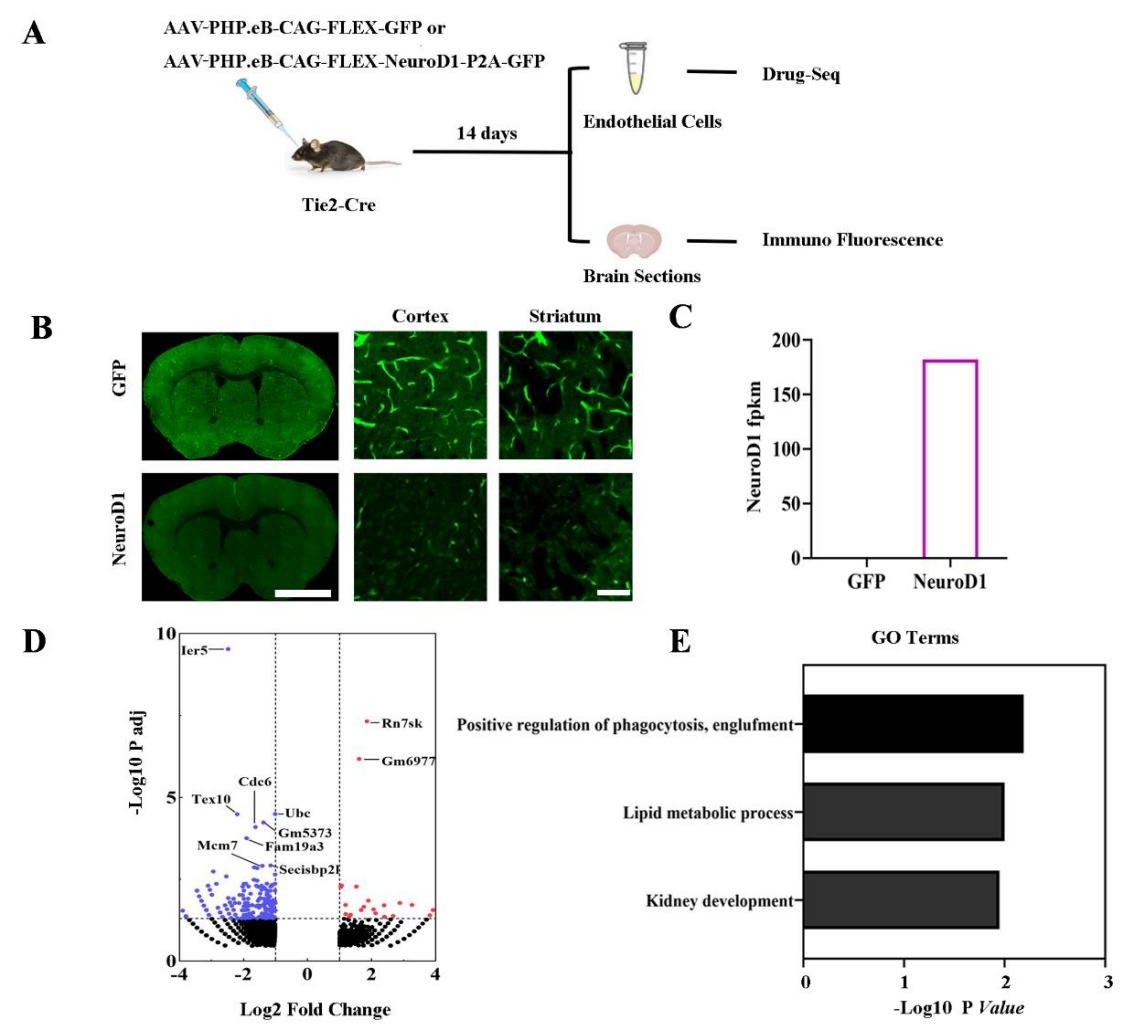
**NeuroD1 modulates AAV-PHP.eB transduction through regulating endothelial gene expression. (A)** Representative schematic of the experimental design Tie2-cre mice subjected to control virus (AAV-PHP.eb-CAG-FLEX-GFP) or NeuroD1 (AAV-PHP.eb-CAG-FLEX-NeuroD1-P2A-GFP) intravenous injection and were sacrificed at 14 days post injection (dpi). Drug-seq was performed to test the gene expression from isolated endothelial cells. Immunofluorensence staining was performed to examine the viral transduction. **(B)** Typical images (whole view and high magnifications from cortex and striatum area) showing the GFP expression in the brain after intravenous administration of AAV-PHP.eb-CAG-FLEX-GFP (GFP, upper panel) or AAV-PHP.eb-CAG-Flex-NeuroD1-P2A-GFP (NeuroD1, lower panel). n = 3 in each group. **(C)** Quantitative analyses of NeuroD1 transcript expression revealed by ffragments per kilobase of exon model per million mapped fragments (FPKM) through Drug-seq. **(D)** Volcano plot showing the differentially expressed genes (DEGs) in response to intravenous administration of NeuroD1 at 14 dpi (adjusted *P* < 0.05, fold change > 2, compared with control mice injected with GFP. The up-regulated and down-regulated DEGs were labeled in red or blue, respectively. Top DEGs were highlighted in box. **(E)** Top 3 Gene Ontology terms of the regulated DEGs upon NeuroD1 administration compared with control GFP samples.

## RESULTS

### Retention of AAV-PHP.eB inside blood vessels after MCAO was reversed by NeuroD1 delivery

To develop non-invasive gene therapy for ischemic stroke, we employed two commonly used BBB-penetrating AAV vectors (AAV-PHP.eB and AAV9) to deliver desired genes [25]. Using the ubiquitous promoter EF-1α to drive reporter gene GFP expression (AAV-PHP.eB-EF1α-GFP or AAV9-EF1α-GFP) in brain cells of adult C57/B6 mice, we intracranially injected the viruses into the striatum and examined GFP expression by immunostaining at 7 days post-viral injection (dpi). The results showed that most of the GFP-transduced cells were NeuN+ neurons in the striatum (Supplementary Fig. 1), suggesting that in normal adult mouse brains, the EF-1α promoter primarily drives the expression of GFP in neuronal cells when directly injected into the brain. Then, we administered AAV-PHP.eB-EF1α-GFP (Fig. 1A) or AAV9-EF1α-GFP intravenously through retro-orbital injection. At 7 dpi, in the case of AAV9-EF1α-GFP (2.6×10^11^ GC/mouse), GFP-positive cells could rarely be detected in the brain (Supplementary Fig. 2). However, in AAV-PHP.eB-EF1α-GFP-treated mice, we found broad AAV transduction in the whole brain, as indicated by GFP-positive cells detected in most brain regions, including the cortex and striatum (Fig. 1B; 7 dpi), suggesting a high penetration efficiency of AAV-PHP.eB than AAV9 in the adult mouse brains. Next, we characterize the AAV-PHP.eB infected cell type following retro-orbital injection and found that while a small number of endothelial cells were infected by the control GFP virus (Fig. 1C, arrow), the majority of GFP+ cells were non-endothelial cells and colocalized with the neuronal marker NeuN (Figure 1D arrowhead). Iba1+ microglia cells and Olig2+ cells were rarely detected among the GFP+ cells (Supplementary Fig. 3A). These results suggest that intravenous delivery of AAV-PHP.eB in normal healthy mouse brains can widely infect the brain and express GFP efficiently in neuronal cells driven by the EF-1α promoter.

Given the role of endothelial cells in AAV-PHP.eB transduction [15-17], we wondered whether AAV-PHP.eB could still cross the BBB efficiently if endothelial cells were severely injured. To answer this question, we employed a transient MCAO model to cause blood vessel damage and then intravenously administered AAV-PHP.eB-EF1α-GFP or AAV-PHP.eB-EF1α-NeuroD1-GFP immediately after reperfusion (Fig. 1E). Unexpectedly, in contrast to the wide infection of the whole brain in normal uninjured mice (Fig. 1B), after ischemic injury, the control virus GFP-expressing cells showed a restrictive pattern in the infarct side (Fig. 1F, left GFP panel, 7 dpi). In contrast, in the NeuroD1-treated mice at 7 dpi, GFP-positive cells were widely distributed, especially outside the infarct areas (Fig. 1F, right NeuroD1 panel). More interestingly, enlarged images revealed that the GFP signal in the control group showed clear morphology of blood vessels in the infarct areas, but in the contralateral side showed morphology of scattered neuronal cells (Fig. 1G, top row). Similar to the non-MCAO mice, the GFP signal was not expressed in IBA1 or Olig2 positive cells in the control virus-treated mice (Supplementary Fig. 3B). In the NeuroD1-treated group, however, GFP signals were found mainly in neuron-like cells in both the peri-infarct areas and on the contralateral side (Fig. 1G, bottom row). Further immunostaining results revealed that a large number of GFP-positive cells in the GFP control group co-stained with CD31, an endothelial cell marker, in the ischemic core region (Fig. 1H, top row), but in the NeuroD1-treated mice, the GFP signal did not co-stain with CD31. Immunostaining with NeuN confirmed that most of the GFP-positive cells in the NeuroD1-treated mice were neurons (Fig. 1I). Quantitative analyses revealed that in normal mice without injury, 97% of the GFP-positive cells were non-endothelial cells, but in GFP-treated ischemic stroke mice, the transduction rate of non-endothelial cells dropped to 52%. However, in the NeuroD1-treated MCAO mice, the transduction rate of non-endothelial cells was reversed back to 99%, as shown in the pie chart (Fig. 1J).

Then, we attempted to determine when the GFP signal could be detected in the endothelial cells (Supplementary Fig. 4), and the results showed that the infected endothelial cells appeared as early as 3 dpi in both the GFP- and NeuroD1-treated mice (Supplementary Fig. 4B). However, there was a significant difference between the two groups when quantifying the transduction rate at 3 dpi: we found that in the GFP-treated mice, 50% of GFP+ cells were co-stained with endothelial cells (Supplementary Fig. 4C) and 50% with other cells, including neurons (Supplementary Fig. 4D) and astrocytes (Supplementary Fig. 4E); in contrast, after NeuroD1 treatment, the ratio of infected endothelial cells dropped to 13%, whereas the infected nonendothelial cells increased to 87% (Supplementary Fig. 4F). Next, we asked how long the GFP signal would stay in the endothelial cells. The results showed that the GFP signal could be detected in the endothelial cells in the control GFP group at two months after MCAO (Supplementary Fig. 4G-H). For NeuroD1 group, the immune-histochemistry results revealed that NeuroD1 was highly expressed in neurons two months after stroke (Supplementary Fig. 4I). Finally, we tested whether endothelial cells could be transduced when AAV-PHP.eB was intracranially administered into the brain after MCAO. We found that when the same vectors (AAV-PHP.eB-EF-1α-GFP or AAV-PHP.eB-EF-1α-NeuroD1-P2A-GFP) were intracranially injected into the brain three days after MCAO, the GFP signal as well as NeuroD1-GFP was mainly present in the neurons 28 days after MCAO (Supplementary Fig. 5). Together, these results suggest that injury of endothelial cells induced by MCAO results in the retention of AAV-PHP.eB in endothelial cells during intravenous gene delivery, which can be reversed by intravenous NeuroD1 gene therapy.

### NeuroD1 regulated the endothelial cells gene expression to modulate transduction of AAV-PHP.eB in Tie2-cre mice

Then, to test whether NeuroD1 has a direct role in modulating AAV-PHP.eB transduction, we engaged Tie2-Cre mice and AAV-PHP.eB-Flex-CAG-NeuroD1-P2A-GFP to restrict the NeuroD1 expression in endothelial cells after DNA recombination. 14 days post intravenous virus injection, the mice were subjected to immunofluoresence staining and endothelial cells isolation for Drug-seq (Fig. 2A). Interestingly, the immunofluoresence results showed that while the GFP signal was strongly observed in the blood vessels in the cortex and striatum area in the GFP control group, GFP signal in the blood vessels was significantly reduced after NeuroD1 treatment (Fig. 2B), suggesting an altered transduction profile. Analyzing the FPKM from Drug-seq showed a dramatic increase of NeuroD1 RNA expression in the endothelial cells after 14 days transduction (Fig. 2C). In addition, NeuroD1-treatment induced 241 DEGs (fold change > 2 or < -2, adjusted *P* < 0.05, FPKM > 0.5) compared to the GFP control group (Fig. 2D). The top 10 DEGs such as Ier5, Rn7sk, Tex10 and Ubc are involved in the DNA damage, epigenetic modulation and transcriptional regulation [26-29]. Furthermore, NeuroD1 also impacted the biological process of phagocytosis in endothelial cells as shown by the GO terms (Fig. 2E). Together, these results suggest that NeuroD1 regulate the transduction profiles of AAV.PHP.eB through modulating the endothelial gene expression and related function.

**Figure 3.**
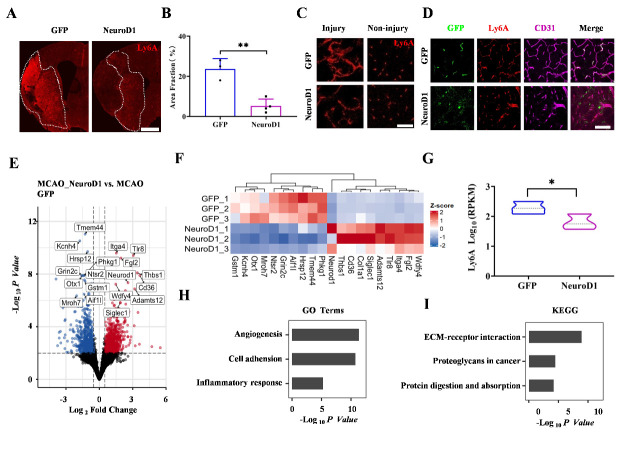
**Intravenous NeuroD1 gene therapy regulates endothelial gene expression after ischemic stroke. (A)** Representative images showing the Ly6A expression in the ipsilateral side of MCAO. Note that Ly6A was significantly upregulated in the infarct area comparing with the peri-infarct area. Dashed lines indicated the infarct zone. Bar = 1 mm. **(B)** Quantitative analyses of the percentage of area covered by Ly6A revealed by immunofluorescence in the ipsilateral side of MCAO mice. n = 3 in the GFP group, n = 4 in the NeuroD1 treated group and was indicated by the dot in the bar graph, each dot represents one animal. Two-tailed unpaired t test. **P < 0.01. **(C)** High magnification images showing the Ly6A expression in the injury site (left) and non-injury site (right) after intravenous administration of AAV-PHP.eB-EF1α-GFP (GFP, upper panel) or AAV-PHP.eB-EF-1α-NeuroD1-GFP (NeuroD1, lower panel). **(D)** Representative images showing that the AAV-PHP.eB-EF1α-GFP transduced cells (GFP, green) were Ly6A (red) and CD31 (magenta) double-positive endothelial cells in the infarct area of MCAO mouse brains (GFP, upper panel). However, the co-localization of transduced cells and endothelial cells was not observed in mice with AAV-PHP.eB-EF-1α-NeuroD1-GFP injection (NeuroD1, lower panel) despite the existence of Ly6A/CD31 double-positive endothelial cells. Samples were at 7 dpi. Bar = 50 μm. **(E)** Volcano plot showing the differentially expressed genes (DEGs) in response to intravenous administration of NeuroD1 at 7 dpi (adjusted *P* < 0.05, fold change > 2, compared with control mice injected with AAV-PHP.eB-EF-1α-GFP). The up-regulated and down-regulated DEGs were labeled in red or blue, respectively. Top DEGs were highlighted in box. **(F)** A heatmap showing the top 10 up-regulated and down-regulated DEGs between NeuroD1-treated and control group. Red or blue color indicates high or low expression level, respectively. **(G)** Quantitative analyses of Ly6A transcript expression revealed by Reads Per Kilobase Per Million (RPKM) through RNA-seq. n = 3 in each group and was indicated by the dot in the bar graph, each dot represents one animal. One-tailed unpaired t test. *P < 0.05. **(H-I)** Top 3 Gene Ontology (GO, H) and KEGG (I) terms of the regulated DEGs upon NeuroD1 administration compared with control GFP samples.

**Figure 4.**
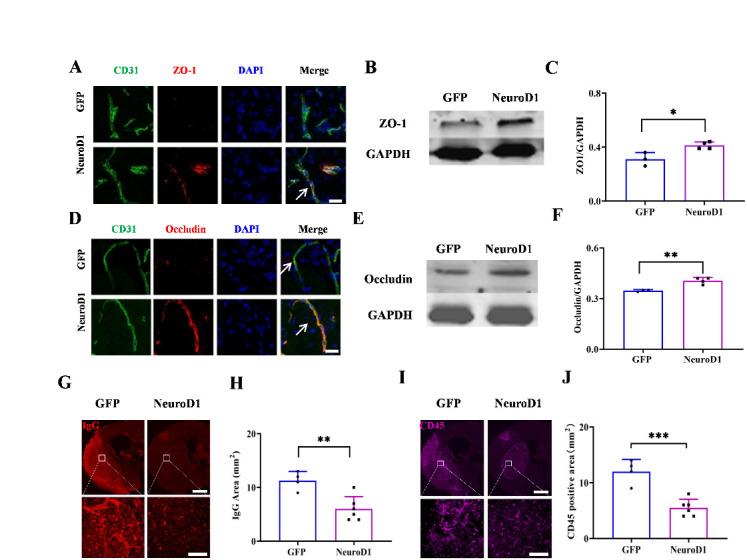
**Intravenous delivery of NeuroD1 preserves BBB integrity after ischemic stroke. (A)** Images showing increased ZO-1 expression (red) in endothelial cells (CD31, green) upon NeuroD1 administration (lower panel) compared with control group (upper panel). Samples were at 7 dpi. Bar = 20 μm. **(B-C)** Western blot image (B) and quantification (C) of ZO-1 expression from cortical tissues at 7 days post MCAO and viral transduction. n = 3 in the GFP group, n = 4 in the NeuroD1 group and were indicated by the dots in the bar graph, each dot represents one animal. Two-tailed unpaired t test. *P < 0.05. **(D)** Images showing that increased Occludin expression (red) in endothelial cells (CD31, green) upon NeuroD1 administration (lower panel) compared with control group (upper panel). Arrowheads indicated the Occludin positive blood vessels. Samples were at 7 dpi. Bar = 20 μm. **(E-F)** Western blot image (E) and quantification (F) of Occludin expression from cortical tissues at 7 days post MCAO and viral transduction. n = 3 in the GFP group, n = 4 in the NeuroD1 group and were indicated by the dots in the bar graph, each dot represents one animal. Two-tailed unpaired t test. **P < 0.01. **(G-J)** Representative low (upper panel) and high (lower panel) magnification images showing the immunostaining of IgG (red, G) and CD45 (magenta, I) in control (left) and NeuroD1-treated (right) group. Bar = 1 mm for the upper panel images and 50 μm for the lower panel images. Quantitative analyses of the area covered by IgG (H) and CD45 (J) revealed by immunofluorescence in the ipsilateral side of MCAO mice. n was indicated by the dot in the bar graph, each dot represents one animal. Two-tailed unpaired t test. **P < 0.01, ***P < 0.001.

### NeuroD1 regulated endothelial gene expression to modulate transduction of AAV-PHP.eB and recovery progress after ischemic stroke

Previous studies have reported that AAV-PHP.eB failed to cross the BBB when it was intravenously injected into Ly6A knockout mice, suggesting a critical role of endothelial Ly6A in guiding AAV-PHP.eB to cross the BBB [17]. Given our finding that AAV-PHP.eB control virus transduction was highly restricted within blood vessels after MCAO but was released into the brain parenchyma after NeuroD1 treatment, we investigated whether NeuroD1 treatment had any direct effect on Ly6A expression. We discovered that after MCAO, the Ly6A expression level was dramatically increased in the infarct areas in the control GFP group (Fig. 3A-B, GFP group). However, in the NeuroD1-treated group, the Ly6A expression level was greatly reduced compared to that in the GFP-treated mice at 7 dpi (Fig. 3A-B, NeuroD1 group). More importantly, enlarged images revealed that the Ly6A expression pattern in the infarct area was drastically different from that in the non-injured site in both GFP- and NeuroD1-treated mice (Figure 3C). Co-expression of GFP with Ly6A and CD31 revealed that control AAV-PHP.eB-GFP-infected cells were largely in blood vessels, but AAV-PHP.eB-NeuroD1-GFP-infected cells did not co-localize with Ly6A or CD31 (Fig. 3D), suggesting that AAV-PHP.eB-NeuroD1-GFP regulated endothelial Ly6A expression to modulate transduction post ischemic stroke.

We further want to understand the impact of NeuroD1 on the global gene expression profile related with recovery of ischemic stroke. We extracted total mRNA from the stroke infarct cortex (7 dpi, 3 replicates for both the NeuroD1 and control groups) and conducted RNA-seq analysis. We first performed pairwise differential expression analysis and divided the differentially expressed genes (DEGs) into upregulated DEGs (fold change > 2, read mean > 80, adjusted *P* < 0.05) and downregulated DEGs (fold change < -2, read mean > 80, adjusted *P* < 0.05) between GFP- and NeuroD1-treated mice. We found that NeuroD1 overexpression induced a total of 701 DEGs (444 upregulated, 267 downregulated) compared to the GFP control group. Volcano plots graphically highlighted the DEGs that were significantly upregulated (red) or downregulated (blue) in response to NeuroD1 treatment (Fig. 3E). Interestingly, most of the top 10 upregulated DEGs, such as Thbs1, CD36, ITGA4, and ADAMTS12, are involved in the function of endothelial cells, as shown in the heatmap (Fig. 3F) [30-33], implying a role of NeuroD1 in regulating the gene expression of endothelial cells post ischemic stroke. The Reads Per Kilobase Million (RPKM) from the RNA-seq data confirmed that Ly6A at the mRNA level was significantly downregulated in the NeuroD1-treated mice compared to the GFP-treated mice (Fig. 3G). After analyzing the overall DEG changes, we examined the detailed biological processes of the DEGs. Using DAVID Bioinformatics, we identified the signaling and biological pathways altered byNeuroD1 gene therapy. Interestingly, the top three biological process-related genes annotated with GO terms were associated with angiogenesis, cell adhesion, and inflammatory response (Fig. 3H). We also used KEGG enrichment analysis of DEGs to characterize their respective biological functions. The most significantly enriched pathway revealed through KEGG enrichment analysis was the ECM-receptor interaction pathway (Fig. 3I), highlighting a role of NeuroD1 in modulating recovery progress after ischemic stroke [34]. Together, these results suggest that intravenous NeuroD1 delivery regulates AAV-PHP.eB transduction and recovery progress after ischemic stroke highly through modulating endothelial gene expression.

### NeuroD1 reduced BBB leakage and attenuated neuroinflammation after MCAO

Endothelial cells possess a series of unique property in sustaining the structure and function of the BBB either through their own tight junctions or in contact with astrocyte endfeet. Our results showed the role of NeuroD1 in regulating endothelial cells gene expression after a transit ectopic expression, we further wonder the effect of NeuroD1 on BBB integrity. We tested the cell junction protein ZO-1, which plays an essential role in BBB integrity by regulating the tight junction among endothelial cells [35]. Immunofluorescent staining results showed that in the GFP-treated infarct areas, ZO-1 expression was largely absent from the CD31-labeled blood vessels, whereas NeuroD1 treatment significantly rescued ZO-1 expression along with CD31-positive blood vessels (Fig. 4A). Western blot analysis of the ipsilateral cortical tissue at 7 days post stroke also revealed that ZO-1 expression was significantly increased after NeuroD1 treatment compared to the GFP control group (Fig 4B-C). We further tested another endothelial-specific tight junction protein, Occludin, using immunostaining (Fig. 4E) and Western blot analysis (Fig. 4D). Similar to the findings on ZO-1 expression, Occludin expression was also significantly increased after NeuroD1 treatment (Fig. 4H). BBB dysfunction after ischemic stroke leads to the leakage of IgG and mononuclear cells from blood vessels into the parenchyma of the brain. To investigate BBB leakage after stroke, we performed immunostaining for IgG and the leukocyte marker CD45. Compared to the GFP control group, the NeuroD1-treated group showed a significant reduction in the IgG-positive signal (Fig. 4I-J) and the CD45-positive signal (Fig. 4K -L). Together, these results indicate that NeuroD1 treatment protects BBB integrity and attenuates BBB leakage post ischemic stroke.

**Figure 5.**
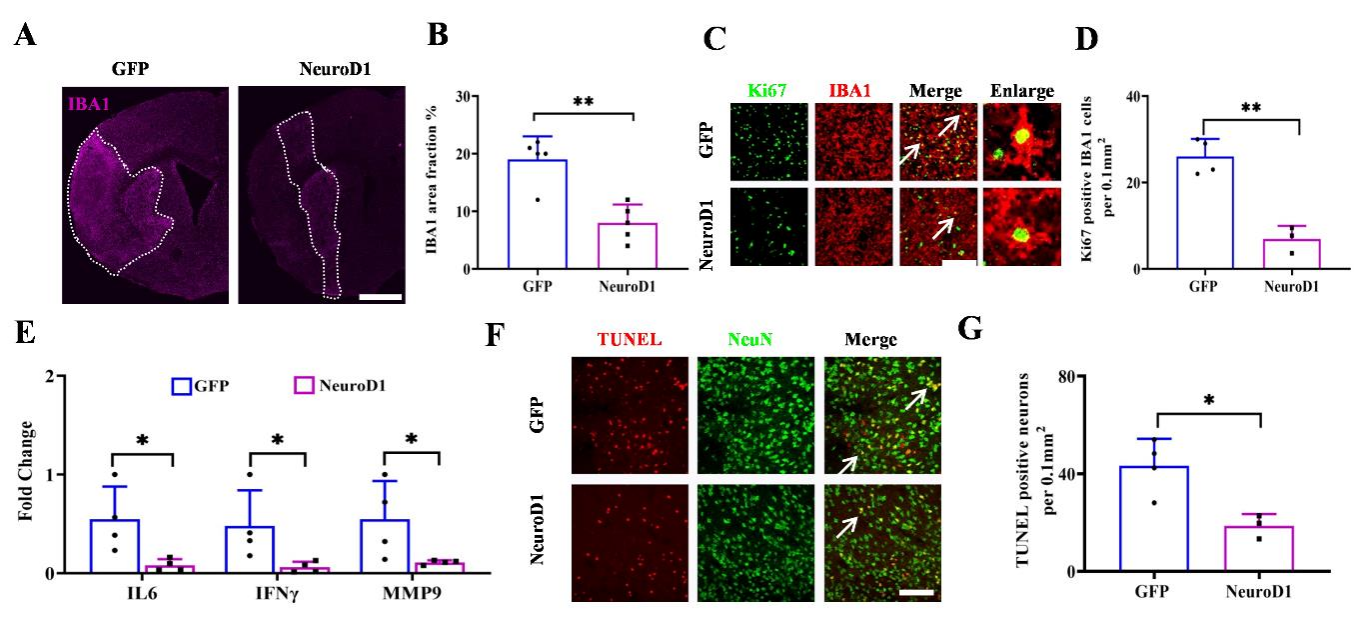
**Intravenous delivery of NeuroD1 inhibits neuro-inflammation and prevents neuron apoptosis after MCAO. (A)** Representative low magnification images showing elevated IBA1 expression (magenta) in the ipsilateral side of the MCAO mice. Dashed lines indicated the infarct zone. Bar = 1mm. **(B)** Quantitative analyses of the percentage of area covered by IBA1 was reduced after intravenous delivery of NeuroD1 compared with control condition. n = 4 in GFP group, n = 5 in NeuroD1 group and were indicated by the dots in the bar graph, each dot represents one animal. Two-tailed unpaired t test. **P < 0.01. **(C)** Representative images showing proliferating microglia (Ki67, green; IBA1, red) in control (upper panel) and NeuroD1-treated (lower panel) group. Arrowheads indicated the Ki67/IBA1 double-positive proliferating microglia. Bar = 50 μm. **(D)** Quantitative analyses showing a reduction of proliferating microglia upon NeuroD1 treatment compared with control group. n = 4 in GFP group, n = 3 in NeuroD1 group and were indicated by the dot in the bar graph, each dot represents one animal. Two-tailed unpaired t test. **P < 0.01. **(E)** Decreased expression of pro-inflammatory molecules (IFN-γ, IL6, and MMP9) upon NeuroD1 treatment compared with control group. n = 3 in each group and were indicated by the dot in the bar graph, each dot represents one animal. Two-tailed unpaired t test. *P < 0.05. **(F-G)** Representative images (F) and quantification (G) showing reduced neuronal apoptosis indicated by immunostaining of TUNEL (red) and NeuN (green). Arrowheads indicated TUNEL/NeuN double-positive apoptotic neurons. n = 4 in the GFP group, n = 3 in the NeuroD1 group and were indicated by the dot in the bar graph, each dot represents one animal. Two-tailed unpaired t test. *P < 0.05.

Neuroinflammation is another pathological phenotype post BBB opening in ischemic stroke pathophisiology. We therefore investigated the effect of the intravenous delivery of NeuroD1 on neuroinflammation after stroke. We found that IBA1+ microglial cells filled the infarct areas in the GFP control group, but in the NeuroD1 group, the IBA1 signal was greatly decreased (Fig. 5A). Quantitative analysis revealed that IBA1+ areas shrank more than half in the NeuroD1-treated group compared to the GFP-treated group (Fig. 5B). We then performed immunostaining for Ki67 to examine proliferative microglial cells after stroke and found a large number of Ki67+ microglial cells in the GFP group, but in the NeuroD1 group, the number of proliferative microglial cells was significantly decreased (Fig. 5C-D). Inflammatory cells in the brain release cytokines that initiate neuroinflammation. We next employed real-time PCR to analyze the expression of cytokines after stroke. We found that proinflammatory factors, including IL6, MMP9, and IFN-γ, were all downregulated in the NeuroD1-treated mice (Fig. 5E). Furthermore, the cell death assay revealed many TUNEL-positive dying neurons in the GFP group, whereas in the NeuroD1 group, neuronal apoptosis was greatly reduced at 7 days after MCAO (Fig. 5F-G). Together, these results suggest that NeuroD1-based intravenous gene therapy attenuates neuroinflammation and inhibits neuronal apoptosis after ischemic stroke.

We next investigated the impact of the intravenous delivery of NeuroD1 on astrocytes response after ischemic stroke (Supplementary Fig. 6A). Immunostaining for GFAP revealed a large infarct area lacking the GFAP signal in the control GFP-treated mice at 7 dpi, but this GFAP-lacking area was greatly reduced after NeuroD1 treatment (Supplementary Fig. 6B-C). Ki67 and GFAP double staining showed that the proliferation of astrocytes was also significantly increased in NeuroD1-treated mice (Supplementary Fig. 6D-E). Interestingly, when we profiled the markers specific for the A1 and A2 subtypes of astrocytes using RNA-seq [36], we found that A1 astrocyte-specific genes were not detected among the DEGs. In contrast, the A2 astrocyte-specific gene EMP1 was upregulated after NeuroD1 treatment (Supplementary Fig. 6F). It was reported that A1 astrocytes are toxic and A2 astrocytes are beneficial [36]. If so, the increase in activated A2 astrocyte gene expression in NeuroD1 group might be protective after ischemic injury.

We and others have demonstrated that NeuroD1 overexpression converts astrocytes into neurons both in vitro and *in vivo* [8, 37]. In the current study, astrocytes were transduced with AAV PHP.eB-EF1α-NeuroD1-GFP at 3 days post-MCAO. We asked whether this NeuroD1 expression can also convert astrocytes into neurons. To test this possibility, we used the Cre-FLEX system to trace astrocytes using the red fluorescent protein mCherry (Supplementary Fig. 6G). In this system, mCherry alone was expressed under the CAG promoter after DNA recombination in astrocytes. Two months post-virus injection (Supplementary Fig. 6H), immunostaining revealed that NeuroD1-GFP-infected cells co-stained with mCherry and NeuN (Supplementary Fig. 6I), indicating that NeuroD1 can convert mCherry-labeled astrocytes into neurons after ischemic stroke.

**Figure 6.**
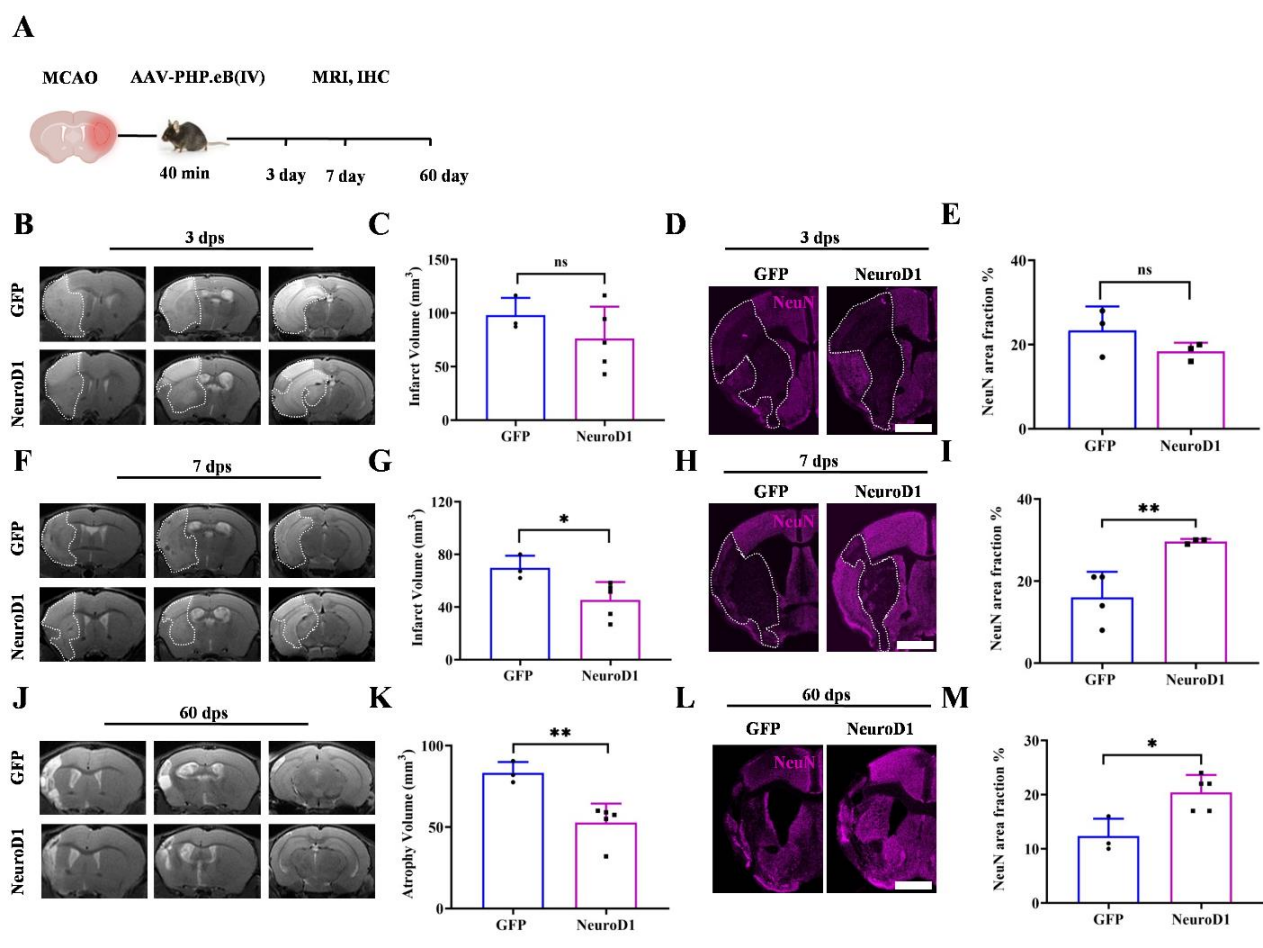
**Intravenous administration of NeuroD1 via AAV-PHP.eB attenuates infarct volume and neuronal loss after MCAO. (A)** Representative schematic of the experimental design. MRI and IHC were performed at the indicated time points to evaluate the infarct volume and neuronal damage respectively. **(B-M)** Representative images of T2-MRI scanning (B, F, J) and NeuN staining (magenta; D, H, I) showing the infarct volume and neuronal loss (B and D, 3 dpi; F and H, 7 dpi; J and L, 60 dpi) in NeuroD1-treated and control group. Dashed lines indicated the infarct zone. Quantification of the infarct volume (C, G,) and atrophy volume (K) indicated by T2-MRI scanning and area covered by NeuN (E, I, M) in NeuroD1-treated and control group (C and E, 3 dpi; G and I, 7 dpi; K and M, 60 dpi). n = 3 in the GFP group, n = 5 in the NeuroD1 group and were indicated by the dots in the bar graph, each dot represents one animal. Two-tailed unpaired t test. *P < 0.05; **P < 0.01; ns = no significance.

### Intravenous delivery of NeuroD1 attenuated infarct size and rescued neuron loss after ischemic stroke

We further used MRI and immunostaining to examine the infarct size and neuronal loss at 3, 7 and 60 days respectively, after MCAO (Fig. 6A). The results from T2-weighted MRI scanning at 3 days after MCAO showed broad injury in the cortex, striatum, and hippocampus in both the GFP- and NeuroD1-treated groups (Fig. 6B). Quantitation of the infarct volume did not show a significant difference between the two groups at 3 dpi (Fig. 6C). Neuronal loss indicated by NeuN staining at 3 dpi confirmed the results from MRI scanning that there was no difference in neuronal loss between the GFP- and NeuroD1-treated groups in early days (Fig. 6D-E). However, at 7 days after MCAO, the infarct volume assessed by MRI revealed a significant decrease in the NeuroD1 group compared to the GFP control group (Fig. 6F-G), and neuronal loss was reduced in the NeuroD1 group, as shown by more NeuN+ signal in the NeuroD1-treated brains than in the GFP-treated brains (Fig. 6H-I). By 60 days post-treatment, the difference was even more striking. Long-term MRI imaging revealed that NeuroD1-treated mice showed smaller infarct volume than GFP-treated mice (Fig. 6J-K). Consistently, the immunostaining results also showed that NeuroD1 treatment significantly rescued neuronal loss at 60 dpi compared to GFP treatment, as shown by a significant increase in the NeuN+ signal in the NeuroD1-treated group (Fig. 6L-M). Together, these results demonstrate that NeuroD1 intravenous gene therapy attenuates ischemic brain injury and rescues neuronal loss after ischemic stroke.

**Figure 7.**
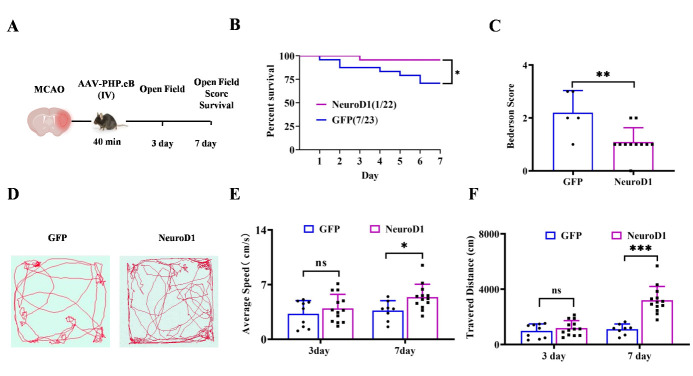
**Reduction of mortality and functional rescue of behavioral deficits after MCAO with NeuroD1 intravenous administration. (A)** Representative schematic of the experimental design to evaluate the behavioral deficits after MCAO and viral transduction. **(B)** Kaplan-Meier survival graph showing the mortality rate was reduced at 7 days post MCAO upon NeuroD1 treatment compared with control group. n was indicated in the graph, Long-rank test. *P < 0.05. **(C)** The Bederson score was significantly decreased in MCAO mice with NeuroD1 administration compared with that in the control group. n = 5 in the GFP group, n = 10 in the NeuroD1 group and were indicated by the dots in the bar graph, each dot represents one animal. Two-tailed unpaired t test, **P < 0.01. **(D)** Representative track plots showing the locomotor activity in the open field test from different groups. **(E-F)** Quantification analyses illustrating the average speed **(E)** and travel distance **(F)** in the open field test. n = 9 in the GFP group, n = 13 in the NeuroD1 group at 3 days, n = 8 in the GFP group, n = 13 in the NeuroD1 group at 7 days and were indicated by the dots in the bar graph, each dot represents one animal, Multiple t test. *P < 0.05; **P < 0.01; ***P < 0.001

### Global NeuroD1 delivery increased survival and promoted behavioral recovery

Next, we investigated whether systemic NeuroD1 delivery would impact neurological behavior after MCAO. We first evaluated the mortality rate in the first 7 days post stroke, as most of the mortality occurred during this time period (Fig. 7A). In the GFP control group, approximately 30% of the mice died in the first week after stroke onset, while the mortality rate in the NeuroD1-treated mice decreased to 4.5%, and only one out of twenty-two mice died from ischemic stroke (Fig. 7B). Then, we performed a modified Bederson score test to assess the severity of brain injury. In this test, a grade scale of 0-5 was used to test forelimb flexion, resistance to lateral push and circling behavior after MCAO. A higher score indicates more severe injury induced by MCAO. The score of NeuroD1-treated mice was significantly lower than that of the surviving GFP-treated mice (Fig. 7C), suggesting that NeuroD1 intravenous gene therapy alleviated MCAO induced behavioral dysfunction at 7 days post-virus injection. In the open field test, which assesses sensory-motor coordination during spontaneous activity, the total travel distance and the average speed were recorded and calculated (Fig. 7D). The data showed that the speed and travel distance were not different between the two groups at 3 days after MCAO (Fig. 7E-F, 3 days); however, the NeuroD1-treated mice traveled faster than the GFP-treated mice at 7 days after MCAO (Fig. 7E, 7 days). The total travel distance of the NeuroD1-treated mice was also significantly longer than that of the GFP-treated mice at 7 dpi (Fig. 7F, 7 days). Together, these results suggest that intravenous NeuroD1 gene therapy can improve functional recovery after ischemic stroke as early as 7 days post-ischemic stroke.

## DISCUSSION

**Figure 8.**
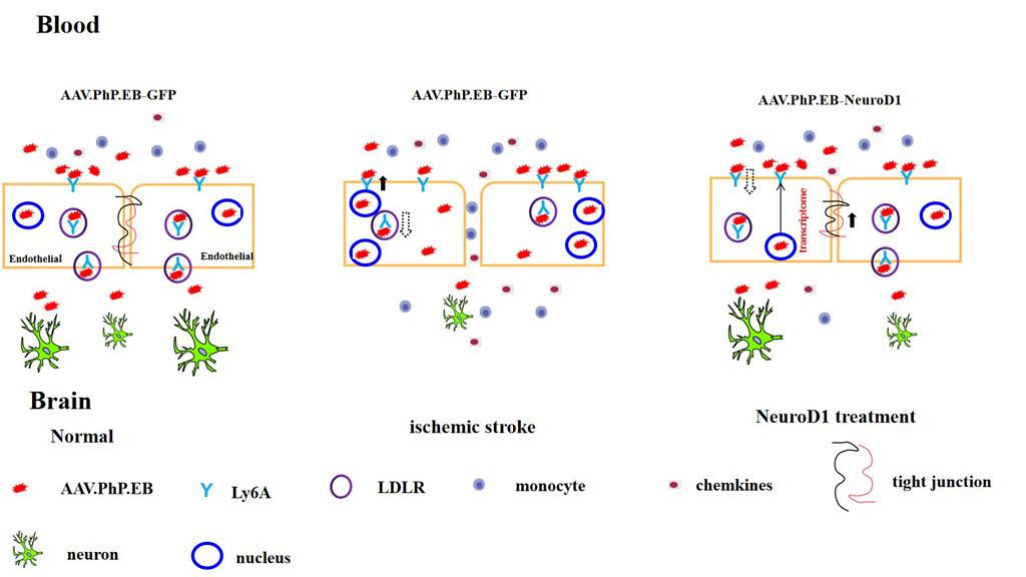
**Models of AAV-PHP.eB entering into the brain post ischemic stroke and NeuroD1 treatment**. Part of AAV-PHP.eB viral particles enters endothelial cell nuclei to mediate transgene expression, while another portion enters brain tissue through transcytosis in the normal brain. AAV-PHP.eB is highly restricted in the endothelial cells post ischemic stroke. NeuroD1 regulates endothelial genes expression to modulate AAV-PHP.eB transduction post ischemic stroke.

The current study highlights a distinctive AAV-PHP.eB spatial-temporal transduction pattern in ischemic stroke mice. Compared with the wide brain cell tropism in the healthy brain, the control virus (AAV-PHP.eB-GFP) was highly restricted inside blood vessels for at least two months after ischemic injury. In contrast, intravenous injection of AAV-PHP.eB carrying therapeutic transgene NeuroD1 (AAV-PHP.eB-NeuroD1-GFP) enhanced wide viral transduction, not limited to the endothelial cells. Moreover, NeuroD1-based intravenous gene therapy after MCAO protected BBB integrity, attenuated neuroinflammation, increased neuronal density, enhanced the survival rate after stroke, and improved behavioral performance. Our study suggests a hypothesis illustrated in Figure 8, proposing a potential progress for AAV-PHP.eB crossing the BBB after an ischemic stroke and post NeuroD1 intravenous gene therapy.

### Transduction profile of AAV-PHP.eB in MCAO mice

The AAV vector employs an active transport mechanism to traverse the BBB, including vector binding and transcytosis. In normal mice, AAV-PHP.eB virus particles bind with Ly6A on the cell surface of endothelial cells and traffic into the brain parenchyma together with the effect of LDLR-APOE mediated transcytosis [15-17, 38]. Ly6A is not only responsible for virus binding but also plays a role in endothelial cell transduction during BBB crossing [18]. Following ischemic injuries, Ly6A expression is upregulated in injured endothelial cells and increases the binding affinity with virus particles, which traps virus particles and enhances endothelial cell transduction. The down-regulated LDLR-APOE signaling pathway (Supplementary Fig. 7) further limited endothelial transcytosis, leading to decreased brain cells transduction in the parenchyma after MCAO. These findings align with a comparative study of AAV transcytosis and transduction using an in vitro BBB model [39]. Furthermore, resent research has showen that AAV-PHP.eB carrying GFP alone triggers significant transcriptome changes in endothelial cells three days after systemic administration in normal mice [40]. Here, we further underscore the the impact of ischemic stroke-induced changes in endothelial gene expression on the transduction efficiency of AAV-PHP.eB. Together, the property and status of endothelial cells play crucial role in determining the efficacy of intravenous gene therapy using AAV-PHP.eB.

Once within brain parenchyma, AAV-PHP.eB infects CNS cells by binding with AAV receptors. In our study, the predominant transduced CNS cell type is neuron under the control of EF-1a promoter even after intracranial injection. This differs slightly from previously reported results, where AAV-PHP.eB transduces neurons, astrocytes and a small number of Olig2-positive cells under the CMV promoter [41, 42]. This difference might be explained by the promoter used in different studies. The EF-1α promoter used in our study drives higher transgene expression in neurons than in other cell types [43].

### Multiple roles of NeuroD1 intravenous gene therapy in reducing neuron loss and promoting stroke recovery

NeuroD1 plays a pivotal role in modulating both central nervous system (CNS) and pancreas development as a transcription factor, regulating the expression of relevant genes [44, 45]. As a pioneer transcription factor, NeuroD1 has the unique ability to access and bind to target DNA sequences (E-box) regardless of chromatin state or cell type [46]. This capacity enables ectopic NeuroD1 expression to initiate gene transcription via an underlying epigenetic mechanism. In our study, even when NeuroD1 was limited in the endothelial cells, we observed significant gene expression up- or down-regulated, suggesting that NeuroD1 can also regulate endothelial gene expression in addition to its role in regulating neuronal gene expression. The precise mechanism underlying how NeuroD1 modulates the endothelial cell gene expression needs to be further investigated.

Endothelial cells, along with other cells including astrocytes and pericytes, form a barrier between the blood and brain tissue termed the BBB, which is critical for the CNS microenvironment. After ischemic stroke, transcriptional changes in endothelial cells contribute to BBB dysfunction, which in turn lead to neuroinflammation and neuronal damage [18]. In the current study, compared to the GFP-treated mice, the total neuronal count was significantly increased after NeuroD1-based gene therapy in MCAO mice. One potential contributing factor might be the creation of a more favorable microenvironment for the survival of pre-existing neurons after NeuroD1 gene therapy treatment. Our previous studies reported that NeuroD1 over-pression in astrocytes protects preexisting neurons from death [3, 4]. Further study showed that NeuroD1 over-expression in astrocytes inhibited neuroinflammation by modulating the properties of reactive astrocytes [6]. In this study, we further demonstrate that NeuroD1 intravenous gene therapy can also repair endothelial injury, protect BBB integrity and inhibit neuroinflammation, creating a conducive microenvironment for neuron survival. The first step during NeuroD1 intravenous gene therapy likely involves the repair of the injured endothelial cells, facilitating subsequent viral particle BBB penetration. More importantly, following the alteration of endothelial gene expression by NeuroD1, the injured brain cell tropism of AAV-PHP.eB was restored, enabling NeuroD1 to further promote stroke recovery through modulating CNS cell’s function.

Through local NeuroD1-based gene therapy, our earlier works on stroke succeeded in increasing the neuron number after endothelin-1 induced focal stroke in mice and rhesus monkeys [3, 4]. A similar effect has also been found in focal MCAO mice by others [47]. In this study, we utilized the GFAP promoter carrying mCherry to track astrocytes and the EF-1a promoter driving NeuroD1 to test its conversion capability. We observed newly regenerated neurons in the injured brains as well following NeuroD1 expression. These results collectively support multiple roles of NeuroD1 intravenous gene therapy in reducing neuron loss and promoting stroke recovery.

### Advantages and limitations of NeuroD1 intravenous gene therapy

Stroke inflicts damages not only on neurons but also on endothelial cells in the neurovascular unit. This study has uncovered that intravenous AAV-PHP.eB-NeuroD-GFP treatment can enhance animal survival rate and behavioral outcomes as early as 7 days post MCAO compared to the GFP treated mice. Mechanically, we found that AAV-PHP.eB carrying NeuroD1 not only repaired endothelial cell injury during BBB penetration but also had a direct effect on neuron regeneration after entering the brain. In this context, gene therapy using AAV-PHP.eB may provide a new systemic therapeutic strategy for acute stroke. Compared to the transgene over-expression before stroke induction [48, 49], NeuroD1 treatment administered post-stroke may be clinically more relevant. From a clinical point of view, it is also of critical importance that NeuroD1 intravenous treatment avoids surgery-related injuries [50]. Given the effect of NeuroD1 on the function of BBB, this novel method has great potential to reduce side effects, such as reperfusion injuries post-stroke.

Despite the advantages of NeuroD1-based intravenous gene therapy for ischemic stroke, some limitations must be kept in mind. First, because of species restriction, Ly6A is not present in primates, which limits the effectiveness of AAV-PHP.eB through systemic injection in humans. Notably, Gradinaru’s laboratory has already developed another serotype (AAV-PHP.V1), which can transduce human endothelial cells, paving the way for potential clinical use [42]. Secondly, besides tMCAO mice, further studies are warranted to test whether such intravenous gene therapy using AAV can be applied to a broader range of stroke models or even other neurological disorders. Lastly, we cannot rule out the impact of this gene therapy on the self-repair mechanisms of ischemic mice. Since viral particles are administered systemically, whether such intravenous gene therapy can affect the self-repair of stroke mice through the actions of other peripheral organs requires further investigation.

It may be worth mentioning that studies reported difficulties in regenerating new neurons from astrocyte lineage-tracing transgenic mice when employing a weak GFAP promoter without enhancer [51, 52]. In contrast, a serial of evidence presented by our recent publications together with other studies clearly demonstrate NeuroD1-mediated astrocyte-to-neuron conversion, including in the astrocyte lineage-tracing mice [8,10,11]. While the academic debate is necessary to clarify disparities in achieving astrocyte-to-neuron conversion among research laboratories, such debates should not serve as a pretext for delaying the pursuit of a potential novel therapy aimed at addressing stroke and other neurological disorders. The newly developed NeuroD1-based intravenous gene therapy provides a novel path toward efficient systemic vascular repair, neuroprotection, and neuron regeneration, thus holding significant potenial for stroke recovery.

## Supplementary Materials

The Supplementary data can be found online at: www.aginganddisease.org/EN/10.14336/AD.2023.1213.

## Data Availability

The raw fastq files and normalized read counts files generated from high-throughout sequencing can be accessed by the number GSE233213 (RNA-seq) and GSE233329 (DRUG-seq). All other datasets generated and/or analyzed during the current study are available from the corresponding author on reasonable request.
