# Supplementary material for: NeuroD1 Regulated Endothelial Gene Expression to Modulate Transduction of AAV-PHP.eB and Recovery Progress after Ischemic Stroke
Source: Aging Dis. 2023 Dec 20;15(6):2632–49. doi: 10.14336/AD.2023.1213 (PMC11567258; doi:10.14336/AD.2023.1213)
Supplement: Supplementary file 1 — The Supplementary data can be found online at: www.aginganddisease.org/EN/10.14336/AD.2023.1213. [file AD-15-6-2632-s.pdf]

SUPPLEMENTARY DATA

NeuroD1 Regulated Endothelial Gene Expression to Modulate Transduction of AAV-PHP.eB and Recovery Progress after Ischemic Stroke

Xiaosong He, Xin Wang, Hui Wang, Tao Wang, Fuhan Yang, Yuchen Chen, Zifei Pei, Yuting Bai, Wen Li, Zheng Wu, Gong Chen

© 2023. He X et al. Published online at http://www.aginganddisease.org/EN/10.14336/AD.2023.1213

SUPPLEMENTARY DATA

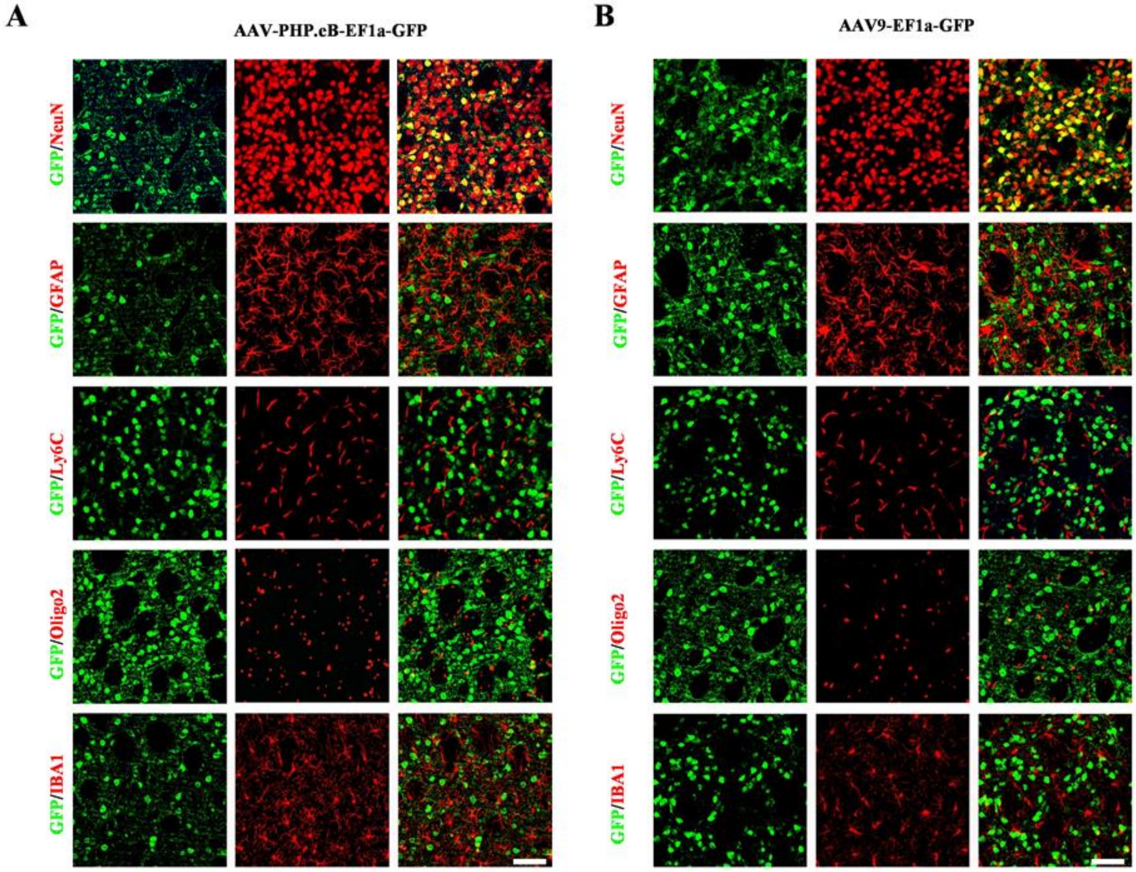

Supplementary Figure 1. AAV-PHP.eB and AAV9 mainly target neurons in normal adult mice via intracranial injection. A-B, AAV-PHP.eB-EF1α-GFP (A) and AAV9-EF1α-GFP (B) mainly transduced neurons (NeuN, red) instead of astrocytes (GFAP, red), endothelial cells (Ly6C, red), oligodendrocyte lineage cells (Oligo2, red), or microglia (IBA1, red) via intracranial injection into the normal adult mouse brains. Bar = 50 μm. Samples were at 7 dpi, n = 3 in each group.

© 2023. He X et al. Published online at http://www.aginganddisease.org/EN/10.14336/AD.2023.1213

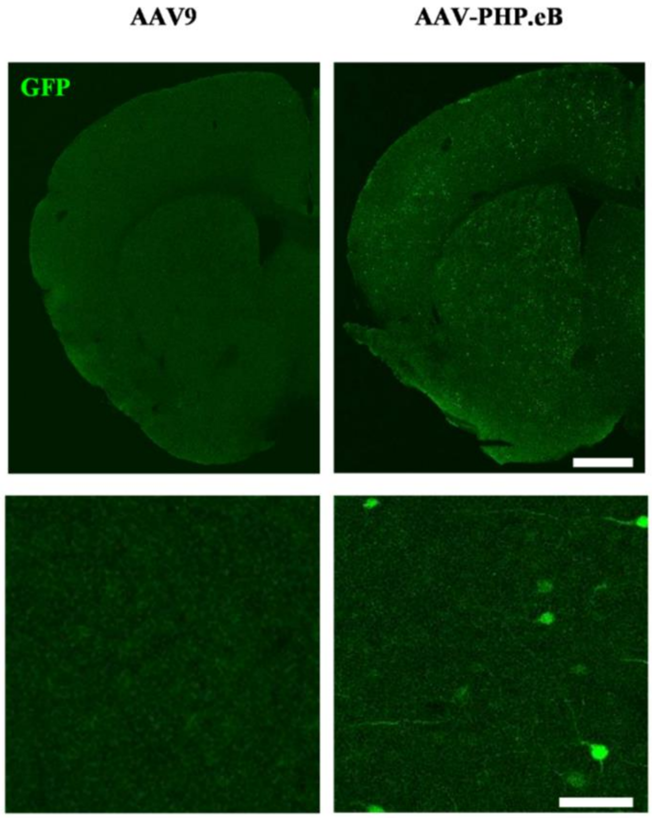

Supplementary Figure 2. Intravenous administration and transduction of AAV-PHP.eB and AAV9 in normal mice. AAV9-EF-1α-GFP failed to cross the BBB at 7 days post virus injection. Note that AA9-EF1α-GFP did not infect endothelial cells. Bar =1 mm and Bar = 50μm in the enlarged figures.

© 2023. He X et al. Published online at http://www.aginganddisease.org/EN/10.14336/AD.2023.1213

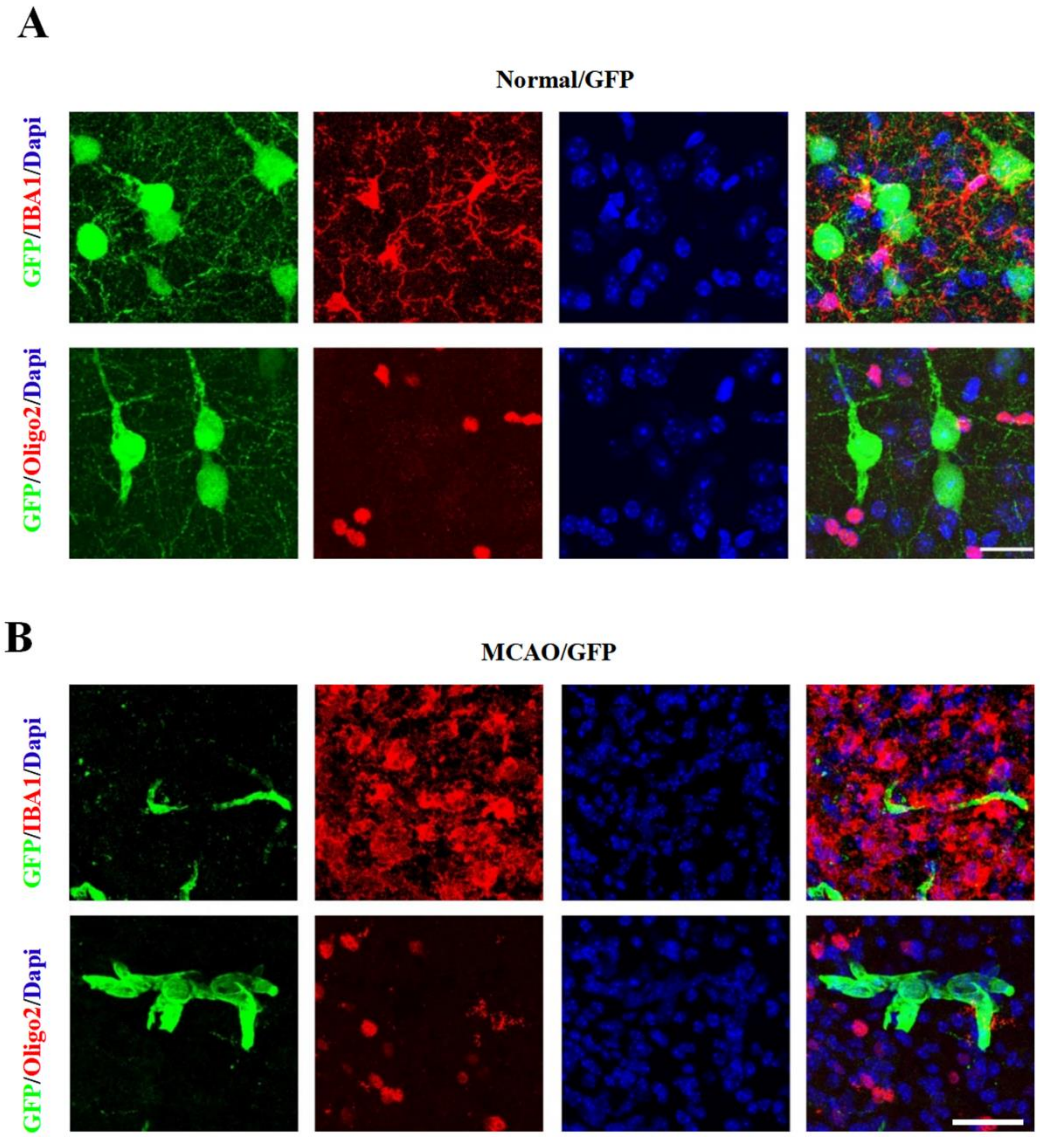

Supplementary Figure 3. AAV-PHP.eB-EF1α-GFP failed to transduce microglia and Oligo2 positive cells in normal and MCAO mice. A-B, The AAV-PHP.eB-EF1α-GFP transduced cells (GFP, green) were not microglia (IBA1, red) or Oligo2 (red) positive cells in the normal (A) and MCAO mice (B) at 7 dpi. Bar = 20μm. Note that the transduced cells exhibited typical neuronal morphology in normal mice (A), but most of the GFP-positive transduced cells showed the morphology of blood vessels in the MCAO mice (B).

© 2023. He X et al. Published online at http://www.aginganddisease.org/EN/10.14336/AD.2023.1213

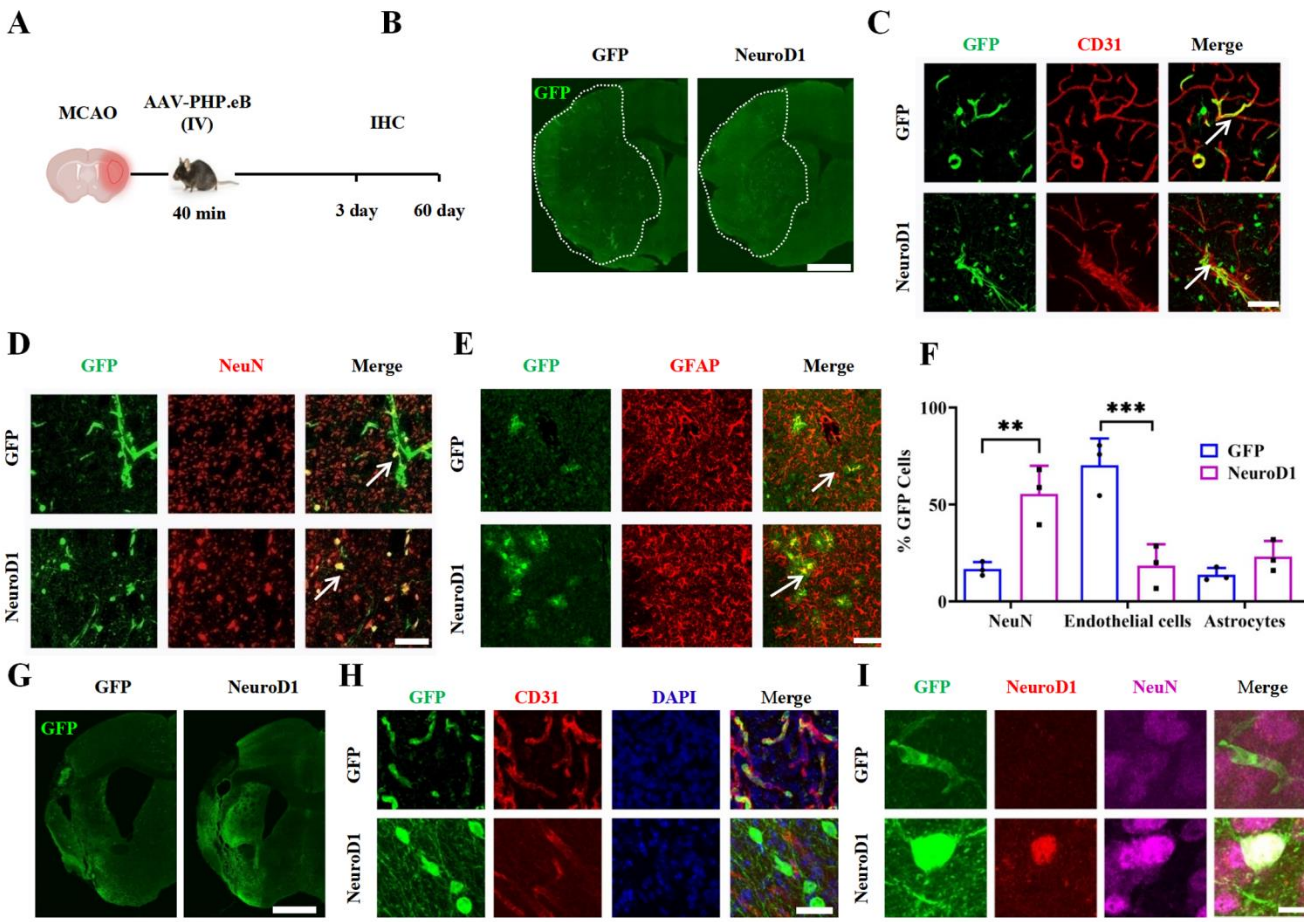

Supplementary Figure 4. AAV-PHP.eB carrying NeuroD1 restores CNS tropism. **A,** Representative schematic showing the experimental design. **B,** Low magnification images showing the viral transduction (GFP, green) in the ipsilateral side of MCAO mice at 3 dpi. Dashed lines indicated the infarct zone. Bar = 1 mm. **C-E,** Representative high magnification images showing that the transduced cells can be CD31 positive endothelial cells (C, arrows), NeuN positive neurons (D, arrows), and GFAP positive astrocytes (E, arrows) at 3 dpi. Note that NeuroD1-expressing AAV-PHP.eB preferentially targeted neurons while control AAV-PHP.eB-EF-1α-GFP mainly targeted endothelial cells. Bar = 50 μm. **F,** Quantification analyses of the transduced cells at 3 dpi. n = 3 in each group and were indicated by the dots in the bar graph, each dot represents one animal. Two-way ANOVA. $^{**}P < 0.01$, $^{***}P < 0.001$. **G,** Sample low magnification images showing the viral transduction (GFP, green) in the ipsilateral side of MCAO mice at 60 dpi. **H-I,** High magnification images showing that some transduced cells (GFP, green) in mice injected with control AAV-PHP.eB-EF-1α-GFP but not AAV-PHP.eB-EF1α-NeuroD1-GFP still exhibited CD31 positive IHC signal (red, H) two months post virus injection. However, in NeuroD1-treated mice, the transduced cells (GFP, green; NeuroD1, red; I) were mainly neurons (NeuN, magenta, I). Bar = 20 μm in H and 10 μm in I.

© 2023. He X et al. Published online at http://www.aginganddisease.org/EN/10.14336/AD.2023.1213

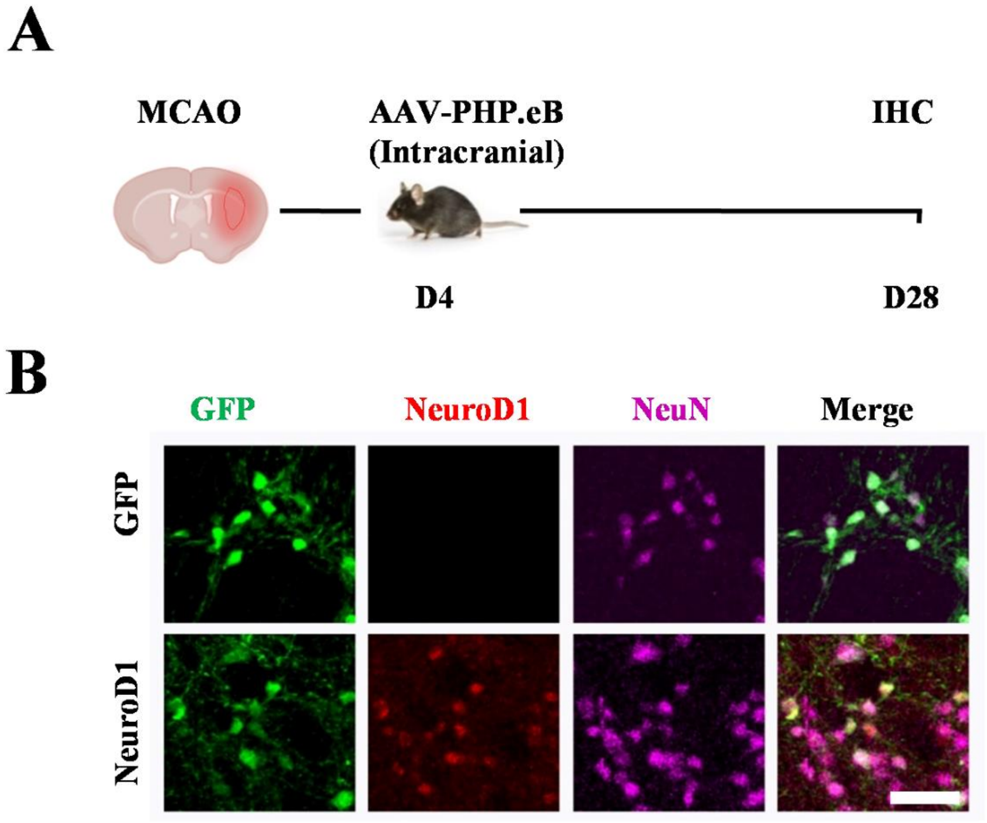

Supplementary Figure 5. AAV-PHP.eB mainly targets neurons with intracranial injection post-MCAO. A, Representative schematic showing the experimental design. Viral particles were stereotactically injected into the ischemic side of the mice 4 days post MCAO, and the tissue samples were collected for observation 28 days after MCAO. **B,** Sample high magnification images showing the transduction (GFP, green) at 28 days postMCAO. Both AAV-PHP.eB-EF1α-GFP (upper panel) and AAV-PHP.eB-EF1α-NeuroD1-GFP (lower panel) mainly target neurons (NeuN, magenta).

© 2023. He X et al. Published online at http://www.aginganddisease.org/EN/10.14336/AD.2023.1213

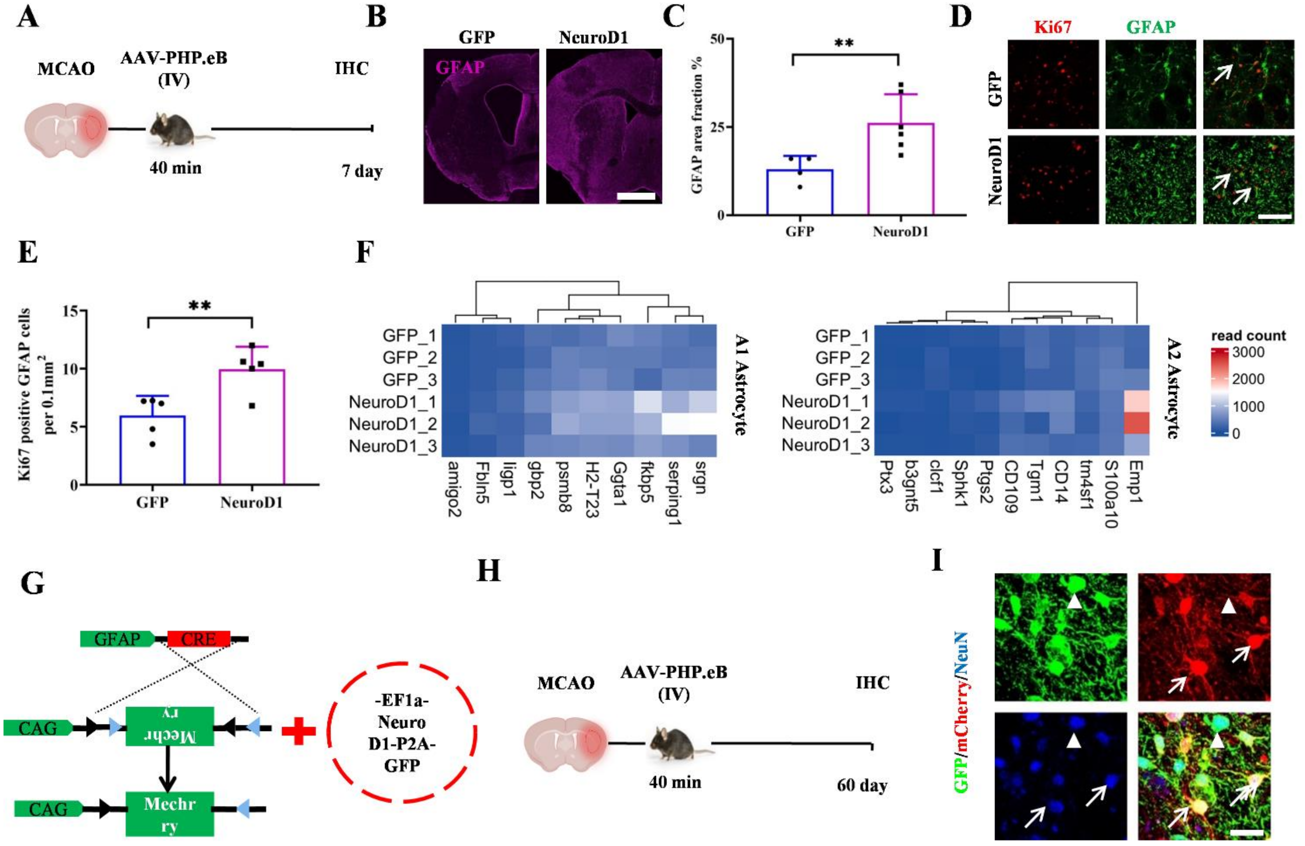

Supplementary Figure 6. Astrocytes response after NeuroD1 intravenous gene administration in MCAO mice. A, Representative schematic showing the experimental design. **B,** Sample images showing GFAP expression (magenta) in the ipsilateral side of the MCAO mice. Bar = 1 mm. **C,** Quantification of the area covered by GFAP. n was indicated by the dot in the bar graph, each dot represents one animal. n = *4* in GFP group, n = 6 in NeuroD1 group and were indicated by the dots in the bar graph, each dot represents one animal. Two-tailed unpaired t test. $**P < 0.01$. **D,** Sample images showing proliferating astrocytes examined by immunostaining of Ki67 (red) and GFAP (green). Arrows showed the proliferating astrocytes. Bar = 50 µm. **E,** Quantification of proliferating astrocytes suggests a significant increase with NeuroD1 overexpression. n = *5* in GFP group, n = *5* in NeuroD1 group and were indicated by the dots in the bar graph, each dot represents one animal. Two-tailed unpaired t test. $**P < 0.01$. **F,** A heatmap showing the expression of A1 and A2 astrocyte signature genes from RNA-seq in NeuroD1-treated and control group. Note that A2 astrocytes marker Emp1 and CD109 were significantly increased by NeuroD1. **G,** Schematic illustrating the AAV-PhP.eB-FLEX-mCherry/AAV-PhP.eB-GFAP::Cre system to pre-label astrocytes after MCAO. AAV-PHP.eB-EF-1α-NeuroD1-GFP were co-injected into the mice to test whether NeuroD1 driven under EF-1α promoter could convert reactive astrocytes into neurons. **H,** Schematic showing the experimental design. **I,** Prelabeled astrocytes transduced by AAV-PHP.eB-EF-1α-NeuroD1-GFP can be converted into neurons, which are positive for GFP (Green), mCherry (Red), and NeuN (Blue) and indicated by arrow. The triangle indicated transduced endogenous neurons that expressed GFP (green) and NeuN (blue), but failed to express mCherry. Bar = 20µm.

© 2023. He X et al. Published online at http://www.aginganddisease.org/EN/10.14336/AD.2023.1213

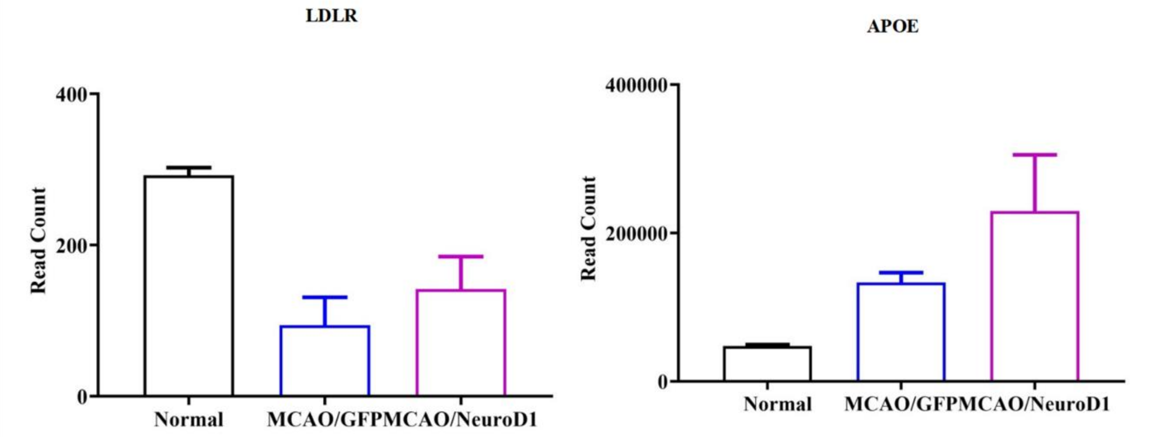

Supplementary Figure 7. LDLR-APOE signal pathway was involved AAV-PHP.eB transdcution after ischemic stroke. A-B, Quantification of LDLR (A) and APOE (B) transcript expression revealed by reads counts through RNA-seq.

© 2023. He X et al. Published online at http://www.aginganddisease.org/EN/10.14336/AD.2023.1213
